# Personals

**Published:** 1913-06

**Authors:** 


### PERSONALS.

*Announcements of removals, travel, and other matters of interest are requested. Please report any errors in the listing of any physician, in the State and other directories that we may co-operate with the State Society in securing a correct list of physicians.*

Dr. Nelson G. Russell of Buffalo is absent on a European trip.

Dr. Ulysses B. Stein, Dr. F. C. Busch and Dr. Thomas J. Walsh of Buffalo have recently returned from Europe.

Dr. George B. Stocker has moved to 855 Genesee street, Buffalo.

We recently had the pleasure of meeting our editorial confrere, Dr. Wm. A. Young of Toronto, at the Chalfonte, Atlantic City.

Dr. Herbert Upham Williams of Buffalo has been appointed State Examiner in Bacteriology, vice Dr. Hiss, deceased.

Drs. Charles G. Stockton and A. L. Benedict of Buffalo attended the meeting of the American Gastro-enterologic Association in Washington, just preceding the Congress.

Dr. Roland O. Meisenbach of Buffalo attended the meeting of Orthopaedic Surgeons at the recent congress in Washington.

Dr. Paul P. A. Preble, U. S. Public Health Service, has recently been detailed to Buffalo for temporary duty in examining samples of lake water, on behalf of the American section of the International Joint Commission.

---

Dr. Walter M. Litchfield of Salamanca was badly injured in an automobile collision, May 8.

---

Dr. Frank E. Howard of Friendship, while assisting at an operation for appendicitis on a patient whom he had taken to Hornell for that purpose, was himself seized with urgent symptoms and was subjected to the same operation, with success, immediately afterward—May 6.

---

Dr. G. S. Peterkin announces his removal of offices to the Cobb Building, Seattle, Wash. His practice is limited to Urology.

---

Dr. Jacob S. Otto of Buffalo has removed his office to 469 Franklin street and his residence to 1094 Delaware avenue.

---

Dr. H. H. Glosser, who succeeded to the practice and the office of the late Dr. Julius Pohlman, has removed to 448 Franklin street, Buffalo.

---

Dr. E. L. Bebee has removed to 401 Elmwood avenue, Buffalo.

---

Dr. Landauer has removed his office to 34 S. Union street, Rochester. Practice confined to Dermatology.

---

Dr. Sherman Voorhees of Elmira, having been obliged to postpone his trip to Germany, expects to leave some time this month.

---

Dr. George M. Gould is taking a much needed rest in Atlantic City.

---

Dr. James F. MacPherson of San Diego, formerly of Tonawanda, has recently spent a month in his former home.

---

Dr. James W. Markoe received a legacy of $25,000 a year from the late James Pierpont Morgan. It is strange that so few physicians receive such acknowledgment of their services.

---

Dr. J. A. Tompkins, Asst. Surgeon, U. S. N., has recently succeeded Dr. H. D. Wilson in charge of the Buffalo recruiting station, who has been ordered to the battleship New Jersey.

The automobile of Dr. S. J. Moyer, Jr., of Lockport was struck by an Erie train May 1, but the occupants escaped serious injury.

A run-away horse leaped into the automobile of Dr. J. J. Brown of Buffalo, May 20. One of his little daughters was stunned but not seriously hurt and some damage was done to the auto.

Dr. George L. Brown of Buffalo has returned from a seven weeks' tour through the South.

Dr. George F. Cott of Buffalo has returned after three months spent in Europe.

Dr. W. B. Cochrane of Rochester has moved to 2097 East avenue.

Dr. Charles M. Gardner, of the U. S. Public Health Service, has recently been transferred from Detroit to Buffalo, succeeding Dr. Duncan A. Carmichael, who has been ordered to the Marine Hospital at Vineyard Haven, Mass.

Dr. Harry Mead of Buffalo left May 31, expecting to spend the summer in Europe.

Dr. Henry C. Buswell of Buffalo was married in Philadelphia April 26, to Mrs. George E. Matthews. They have left for a European trip.

